# Impact of Obesity on Acyclovir-Induced Nephrotoxicity

**DOI:** 10.1093/ofid/ofz121

**Published:** 2019-03-07

**Authors:** Katie E Barber, Jamie L Wagner, Kayla R Stover

**Affiliations:** Department of Pharmacy Practice, University of Mississippi School of Pharmacy, Jackson, Mississippi

**Keywords:** acyclovir, antimicrobial stewardship, HSV, nephrotoxicity

## Abstract

**Background:**

Obesity is a major medical issue nationally, with rates continually increasing. In obese patients, minimal data exist for appropriate dosing of acyclovir to decrease the rates of nephrotoxicity. The purpose of this study was to determine the prevalence of and risk factors associated with acyclovir-induced nephrotoxicity.

**Methods:**

A retrospective case–control of patients who received intravenous acyclovir for >48 hours at the University of Mississippi Medical Center over a 4-year period were evaluated to elucidate the prevalence of acyclovir-induced nephrotoxicity. Additionally, risk factors for the development of nephrotoxicity, including the effect of obesity and dosing strategy, were assessed.

**Results:**

One hundred fifteen patients were included in the study. A total of 24 (21%) patients developed nephrotoxicity after acyclovir exposure and were in the Risk (9.6%), Injury (4.3%), and Failure (7%) categories, defined by the RIFLE criteria. Neither acyclovir dosage, fluid status, nor baseline characteristics, other than obesity, varied between those who developed nephrotoxicity vs those who did not. Independent predictors of nephrotoxicity were obesity (odds ratio [OR], 3.2; 95% confidence interval [CI], 1.19–8.67) and receipt of vancomycin (OR, 4.73; 95% CI, 1.57–14.25). No differences in vancomycin dosing or concentrations were observed between the patients who developed nephrotoxicity and those who did not.

**Conclusions:**

In this study, nephrotoxicity occurred in 21% of patients receiving acyclovir. Concomitant vancomycin receipt and obesity led to higher rates of toxicity. Efforts should be made to target obese patients on acyclovir plus vancomycin and discontinue therapy in patients not warranting antiviral coverage to minimize chances of toxicity.

Obesity is a major medical issue nationally, leading to health complications, morbidity, and mortality. National rates indicate that >35% of adults are obese, with states where large portions of the population have low access to care, including Louisiana, Mississippi, Alabama, and West Virginia, each reporting even higher rates [1, 2]. In patients who are obese, clinicians are often challenged regarding how to properly address medical issues, especially with medications that require dosing based upon body weight.

Although some weight-based antimicrobials have recommendations for use of either actual body weight (ABW) or ideal body weight (IBW), many of these drugs have little evidence available to guide clinician dosing. With the increasing rates of obesity and morbid obesity, ABW and IBW are increasingly divergent, prompting many clinicians to perform dosage calculations based upon an adjusted body weight (AdjBW), a calculated value that falls between the IBW and ABW.

Acyclovir, approved in 1982 for the treatment of herpes simplex virus (HSV), is one such drug that is dosed according to weight [3]. A single study evaluated the pharmacokinetics of acyclovir in 7 morbidly obese patients and 5 normal-weight patients and displayed similar pharmacokinetics between groups [4]. Because obese patients displayed comparable pharmacokinetics as normal-weight persons and given data that demonstrate increased dosages resulting in higher rates of nephrotoxicity from 12% to 48% [5–7], the recommendation for IBW dosing was chosen.

Unfortunately, no data exist regarding the rates of nephrotoxicity between the potential dosing weights or the specific cause of nephrotoxicity in the adult population (ie, direct toxicity from acyclovir vs a combination of risk factors). Identification of these risk factors will aid clinicians in providing an evidence-based approach to current clinical practice, thus providing a higher quality of care to all patients receiving acyclovir. The purpose of this study was to determine the prevalence of and risk factors for acyclovir-associated nephrotoxicity in an adult population at the University of Mississippi Medical Center (UMMC).

## METHODS

### Study Design, Setting, and Patient Population

A retrospective case–control study was conducted at UMMC among adult (≥18 years old) patients who received intravenous (IV) acyclovir for >48 hours between January 1, 2014, and December 31, 2014. UMMC is a university-affiliated tertiary hospital and referral center with >700 inpatient beds. Patients were identified through use of clinical decision support software (TheraDoc). Excluded patients were those receiving hemodialysis or other forms of renal replacement therapy before the receipt of acyclovir and those on vasoactive medications. If a patient received more than 1 course of IV acyclovir, subsequent exposures were not included. Included patients were analyzed to determine the frequency of IV acyclovir–associated nephrotoxicity. The patients were divided into 2 groups: those who developed toxicity (cases) and those who did not (controls).

### Study Variables and Definitions

Demographics (patient age, gender, race, weight, and comorbidities), microbiological data, intensive care unit (ICU) status, and presence of infectious diseases consultation were collected. Additional data collected included information pertaining to the primary study end point of nephrotoxicity: weight-based dose, baseline renal function (obtained the day before acyclovir initiation), renal function while on IV acyclovir therapy, new-onset renal replacement therapy, and reduction in or discontinuation of acyclovir. Lastly, known risk factors for nephrotoxicity were also collected, including concomitant nephrotoxic agents (aminoglycosides, vancomycin, angiotensin-converting enzyme inhibitors, and radiocontrast dye) and hydration status (concomitant fluids). Obesity was defined per the World Health Organization as a body mass index (BMI) ≥30. Nephrotoxicity was defined according to the RIFLE criteria, which were calculated with the highest serum creatinine value during acyclovir therapy [8]. In the microbiologically evaluable population (those with punch biopsy–proven or polymerase chain reaction [PCR]–proven viral infection), therapeutic success was assessed via return to baseline mentation for central nervous system (CNS) involvement or reduction in size, frequency, or crusting of lesions for cutaneous infections. Acyclovir dosing was recorded as milligram per kilogram of ABW, AdjBW, and IBW.

### Statistical Analyses

Statistical analysis was performed using SPSS software, version 23.0 (IBM). Categorical data were analyzed using the chi-square or Fisher exact test, and continuous data were analyzed using the Student *t* test or Mann-Whitney *U* test, as appropriate. An alpha of ≤.05 was deemed statistically significant. Variables that had a *P* value <.2 on univariate analysis or that were deemed clinically relevant by the investigators were evaluated for inclusion in a multivariable logistic regression model, and adjusted odds ratios (ORs) with associated confidence intervals (CIs) were calculated. Clinically relevant was defined as variables with prior documentation of contributing to the development of nephrotoxicity.

## RESULTS

A total of 115 patients were enrolled in the study. Patient characteristics are listed in Table 1. The median age (interquartile range [IQR]) was 54 (37–64) years, with a predominately female (52%) population. Among the 115 patients, acyclovir-associated nephrotoxicity, defined by the RIFLE criteria, was observed in 24 (21%) patients. Among those who developed nephrotoxicity, there were 11 (9.6%) in the Risk category (serum creatinine increased 1.5× over baseline), 5 (4.3%) in the Injury category (serum creatinine increased 2× over baseline), and 8 (7%) in the Failure category (serum creatinine increased 3× over baseline). No patients experienced loss of renal function beyond 4 weeks.

**Table 1.** Patient Demographics

	Total Population (n = 115)	Toxicity Group (n = 24)	Nontoxicity Group (n = 91)	*P* Value
Age, y	54 [37–64]	55 [45–66]	52 [35–64]	.39
Male	55 (47.8)	9 (37.5)	45 (50.5)	.26
Comorbid conditions				
Obese	55 (47.8)	16 (66.7)	38 (42.9)	.04
Diabetes	35 (30.4)	6 (25)	29 (31.9)	.52
Dyslipidemia	15 (13)	5 (20.8)	10 (11)	.30
Hypertension	62 (53.9)	16 (66.7)	46 (50.5)	.35
Heart failure	8 (7)	3 (12.5)	5 (5.5)	.36
Chronic kidney disease	12 (10.4)	3 (12.5)	9 (9.9)	.71
Cancer	64 (55.7)	11 (45.58)	53 (58.2)	.28
Renal transplant	1 (0.9)	0 (0)	1 (1.1)	1.00
Concomitant nephrotoxic agents				
ACE inhibitors	17 (14.8)	6 (25)	11 (12.1)	.19
Aminoglycosides	1 (0.9)	0 (0)	1 (1.1)	1.00
Amphotericin	4 (3.5)	1 (4.3)	3 (3.3)	1.00
Contrast dye	27 (23.5)	8 (33.3)	19 (20.9)	.20
Loop diuretics	23 (20)	6 (25)	17 (18.7)	.57
NSAIDs	11 (9.6)	2 (8.3)	9 (9.9)	1.00
Vancomycin	63 (54.8)	19 (79.2)	44 (48.4)	.04
Concomitant fluids	94 (81.7)	21 (87.5)	73 (80.2)	.56
Baseline serum creatinine, mg/dL	0.73 [0.6–1.1]	0.6 [0.5–0.8]	0.8 [0.6–1.1]	.07
Highest serum creatinine, mg/dL	0.98 [0.7–1.3]	1.9 [1.1–3]	0.9 [0.7–1.2]	<.001
Day of highest serum creatinine, mg/dL	4 [2–5]	4.5 [3–7]	3 [1–5]	.003
Admission weight, kg	79.5 [67–98]	89.9 [67–120]	79.1 [67–95]	.18

Data are presented as No. (%) or median [interquartile range].

Abbreviations: ACE, angiotensin-converting enzyme; NSAID, nonsteroidal anti-inflammatory drug.

In univariate analysis, obesity was the only comorbid condition associated with nephrotoxicity (*P* = .04). There were no differences in receipt or type of IV hydration or concomitant nephrotoxic agents, other than receipt of vancomycin. Although 63 (55%) patients received vancomycin, there were no differences between the 2 groups on maximum vancomycin dose (*P* = .76), frequency of initial trough >20 mg/L (*P* = .71), or maximum total daily dosage (*P* = .52). Furthermore, there were no differences between groups in trough concentrations for any troughs obtained throughout vancomycin therapy. Baseline serum creatinine values were similar between groups, with a median initial value of 0.75 mg/dL (*P* = .09). As expected, the patients who developed nephrotoxicity displayed higher serum creatinine values, with the peak values observed at a median (IQR) of day 4.5 (3–7) of acyclovir therapy. Of the patients who developed nephrotoxicity, 4 (17%) needed renal replacement therapy. More patients (50% vs 22%) who experienced nephrotoxicity were admitted to the ICU during their hospitalization compared with control patients (*P* = .01). These patients also had longer stays in the ICU, at a median (IQR) of 10.5 (7–14.8) days vs 5 (3–7.5) days, respectively (*P* = .01). Although total length of stay was comparable between both groups, the length of stay postinitiation of acyclovir therapy was 4 days longer in the patients who developed nephrotoxicity (*P* = .03).

All patients were initiated on thrice-daily dosing of acyclovir. The median total daily dose (IQR) was 1950 (1575–2325) mg, with a median dose of 10.1 (9.9–11.4) mg/kg and 8.1 (6.0–10.2) mg/kg based upon IBW and ABW, respectively. There were no differences observed between either group (*P* = .67). Acyclovir-associated nephrotoxicity was not associated with dosing weight utilized (*P* > .05). Patients who developed nephrotoxicity were treated with acyclovir for a median (IQR) of 8.5 (5.25–11) days, whereas the control group was treated with acyclovir for a median of 6 (5–9) days (*P* = .053).

The majority (62%) of patients initiated on acyclovir therapy had an indication for treatment of a CNS infection. However, only 17 (15%) patients had positive microbiological results, with the most commonly identified organism being HSV. There were no differences between groups in indication for acyclovir, infection site, time to baseline mentation, or time to lesion size reduction and lesion crusting.

The majority (80.9%) of patients were on additional agents known to cause nephrotoxicity, with the most frequently identified concomitant nephrotoxin being vancomycin. Although more patients in the nephrotoxicity group received vancomycin, there were no differences in maximum cumulative daily dosage, maximum total dosage, frequency of dosing, or initial trough values. In multivariate logistic regression, both obesity (OR, 2.67; 95% CI, 1.04–6.86) and receipt of vancomycin (OR, 4.06; 95% CI, 1.40–11.80) were independently associated with increased nephrotoxicity (Table 2).

**Table 2.** Multivariate Analysis for Independent Risk Factors for Acyclovir-Associated Nephrotoxicity

	No. (% Total Population)	Unadjusted OR (95% CI)	Adjusted OR (95% CI)
Obesity	55 (47.8)	2.667 (1.037–6.859)	3.212 (1.191–8.666)
Vancomycin	63 (54.8)	4.059 (1.396–11.804)	4.735 (1.573–14.253)

Abbreviations: CI, confidence interval; OR, odds ratio.

## Conclusions

Approximately one-fifth of the patients included developed acyclovir-associated nephrotoxicity after approximately 5 days of therapy. Although obesity was the only comorbidity difference between the cases and controls, the total daily acyclovir dosage did not differ between the 2 groups. Although many patients received concomitant nephrotoxins, there was an equivalent distribution other than the receipt of vancomycin. However, patients in both groups had comparable vancomycin dosing and trough concentrations. Both vancomycin receipt and obesity were independent risk factors for the development for acyclovir-associated nephrotoxicity, leading to numerically increased length of hospital stay and statistically increased length of stay after acyclovir initiation.

Published rates of acyclovir-associated nephrotoxicity range between 12% and 48% [5, 9, 10]. However, the effect of obesity has not been definitively defined, though several case reports have suggested that weight may be the culprit [11, 12]. Our study demonstrated comparable findings with a 21% rate of nephrotoxicity. Additionally, obesity was an independent predictor of nephrotoxicity in these patients. Unfortunately, there currently is not a consensus on how acyclovir should be dosed in these patients. An abstract presented at the Interscience Conference on Antimicrobial Agents and Chemotherapy in 1991 suggested that morbidly obese patients should be dosed according to IBW based upon excessive acyclovir concentrations in 7 morbidly obese vs 5 normal-weight patients utilizing ABW [4]. Conversely, Turner and colleagues evaluated IBW dosing of acyclovir in 14 patients, 7 of whom were morbidly obese, and determined that systemic exposure in the morbidly obese patients provided substantially lower exposure than that of the nonobese patients [13]. Although obesity was a risk factor for toxicity in our study, we did not observe a difference in the rates of acyclovir-associated nephrotoxicity regardless of weight utilized for dosing calculations.

Our study suggests that, in institutions that frequently utilize IV acyclovir, antimicrobial stewardship should target obese patients on concomitant vancomycin. Only 15% of our patients yielded positive microbiological results, suggesting that acyclovir exposure could have been minimized in this population and that stewardship efforts should be focused on these patients. With HSV PCR results available within 24 hours at many institutions, boasting a sensitivity of up to 90%, acyclovir therapy should be discontinued as long as clinical judgment does not warrant continuation [14]. On January 1, 2017, the Joint Commission released standards recommending antibiotic timeout at 48 hours as part of a targeted antimicrobial stewardship effort [15]. With nephrotoxicity not occurring until an average of 4.5 days after acyclovir initiation, it would be prudent to also assess antiviral agents at that time, particularly in patients receiving concomitant vancomycin.

Although our findings provide more insight into the risk factors for acyclovir-associated nephrotoxicity, there are several limitations. We were unable to find a causative dosing strategy to help aid clinicians in selection of an optimal dose for clinical use. Additionally, we had a small sample in our microbiologically evaluable population, so we cannot suggest improved or worsened efficacy based upon dosing strategy. Furthermore, there were more patients who received vancomycin in the nephrotoxicity group than in the control group, which makes it difficult to discern if the vancomycin or the acyclovir or both were responsible for causing nephrotoxicity. Lastly, although we attempted to be thorough with our data collection, there may be another factor associated with acyclovir-associated toxicity that we did not examine.

Acyclovir-associated nephrotoxicity occurred in 21% of acyclovir-treated patients and was associated with an increased infection-related length of hospital stay after toxicity development. Both obesity and concomitant vancomycin usage were independent factors that increased risk for the development of acyclovir-associated nephrotoxicity. Antimicrobial stewardship efforts should target obese patients on acyclovir plus vancomycin and discontinue therapy in patients not warranting antiviral coverage to minimize toxicity. Further investigations on nephrotoxicity in patients on both vancomycin and acyclovir are warranted.
